# 

**Published:** 1894-03-17

**Authors:** 


**Notice to Advertisers and Subscribers.**

All Advertisements, Orders for Copies of The Hospital, and Business Communications should be addressed to The Publisher, *not to the Editor*, The Hospital, 428, Strand, W.C.

The Cost of Subscription, post free, is as follows:—Per week, 2½d.; per quarter, 2s. 8d.; per half-year, 5s. 3d.; per annum, 10s. 6d. Foreign:—Per Annum, 12s. 8d.; payable, in all cases, in advance.

**OUR NEW OFFICES.**

428, Strand, W.C., will henceforth be the Office of this Journal.

**Notices of Births, Deaths, and Marriages** are inserted in this column at a charge of 2s. 6d. for an announcement not exceeding four lines.

**NOTICE TO CORRESPONDENTS.**

All MS., Letters, Books for Review, and other matters intended for the Editor should be addressed The Editor, The Lodge, Porchester Square, London, W.

The Editor cannot undertake to return rejected MS., even when accompanied by stamped directed envelope.

**"Burdett's Hospital Annual," 1894.**

Fifth year of publication. 700 pp. Boards, gilt lettering. A most complete guide to British, Colonial, Indian, and American Hospitals, Dispensaries, Nursing and Convalescent Institutions, and Asylums. Price 5s. Published by The Scientific Press (Limited), 428, Strand, W.C.

**NOTICE.—The Index for Vol. XIV. (ending Sept. 30th, 1893) is on sale at the Office of this Journal, price 2½d., post free.**

### SCRAPS AND GLEANINGS.

It is satisfactory to notice that although the daily number of in-patients to the Shepton Mallet Hospital throughout the year has increased 9·25, the total expenditure on maintenance of patients is lower than at any period during the last ten years.

Objections continue to be raised to the sites suggested for the Margate and Ramsgate Joint Hospital for Infectious Diseases.

At the annual meeting of the governors of the Children's Hospital, Bradford, the Board deplored the fact that the means placed at their disposal are inadequate to the work they have to perform. The balance-sheet discloses a serious deficit on the accounts.

The work of the Bath Royal United Hospital during the period under review at the recent annual meeting is again marked by the great increase of patients which have been treated in the hospital.

At the annual meeting of the subscribers of the Tunbridge Wells Homœopathic Hospital, it was stated that the institution was worked up to full power, and although no appeal was made, the Committee report that they are anxious to receive donations for a reserve fund, to be used in extending the premises.

The receipts of the Southend Viciori**a** Hospital were reported at the annual meeting to be in excess of the previous year by £28; a decrease in subscriptions being compensated for by donations, one being of the value of £80, the result of a sa e of work at the Alexandra Yacht Club.

The Cheshire County Council have just issued an order to all the sanitary authorities within its district directing them to add phthisis to the list of notifiable diseases under the Notification of Infectious Diseases Act.

### Medical Vacancies and Appointments.

### APPOINTMENTS.

E. W. P. Baines, M.B., appointed House Physician to the Hospital for Women, Soho. S. M. Brown, M.B.Lond., L.R.C.P., appointed House Surgeon to the Salford Royal Hospital. E. A. Browne, F.R.C.S.Edin., appointed Consulting Surgeon to the Liverpool Eye and Ear Infirmary. W. F. Clark, M.R.C.S.Eng., L.S.A., appointed Medical Officer to the Cheshunt District of the Edmonton Union. D. S. Dunn, M.D., M.Ch., L.M., appointed Medical Officer to the Warboy District of the St. Ives Union. B. Edwards, L.R.C.P.Lond., M.R.C.S.Eng., appointed Medical Officer of the Ipswich District of the Samford Union. C. Grey-Edwards, B.A., M.B., B.Ch.Univ.Dubl., appointed Public Vaccinator to the No. 1 Anglesey District of the Bangor and Beaumaris Union. W. A. Hooton, L.R.C.P.Lond., M.R.C.S., L.D.S., appointed Honorary Dental Surgeon to the General Hospital for Sick Children, Manchester. E. Knight, M.R.C.S., L.R.C.P., appointed Junior House Surgeon to the Salford Royal Hospital. H. L. Lack, M.B., F.R.C.S., appointed Demonstrator in Surgery to King's College, London. J. H. Lightbody, M.D. Vict., M.B. and Ch.B., appointed Medical Officer to the Wybunbury District of the Nantwich Union. M. O'Sullivan, M.B.R.U.I., appointed Pathologist and Anæsthetist to the Children's Hospital, Temple Street, Dublin. A. E. Rook, L.R.C.P.Lond., M.R.C.S. Eng., appointed Medical Officer to the Eastbourne Union. H. H. Rubra, M.R.C.S., L.R.C.P., appointed Assistant House Surgeon to the Cumberland Infirmary, Carlisle. C. H. B. Shears, M.R.C.S.Eng., L.R.C.P.Lond., appointed Surgeon to the Liverpool Eye and Ear Infirmary.

### VACANCIES.

The following vacancies are announced this week, the amount of salary in each case being given after the name of the institution:

#### Resident Medical Officers.

Birmingham General Dispensary, Birmingham. Resident Surgeon. Salary £150 per annum, with an allowance for cab hire, and furnished rooms, fire, lights, and attendance. Applications and testimonials to Alex. Forrest, Secretary, by April 11th.

Bolton Infirmary and Dispensary. Junior House-Surgeon; age not to exceed 25. Salary £80 per annum, with furnished apartments, board, and attendance. Applications and testimonials to Peter Kevan, Honorary Secretary, 12, Acresfield, Bolton, by March 20th.

Devonshire Hospital, Buxton, Derbyshire. Assistant House-Surgeon. Salary £50 per annum, with furnished apartments, board, and washing. Applications and testimonials to Joseph Taylor, Secretary, by March 19th.

General Hospital, Birmingham. Two Assistant House-Surgeons. Appointment for six months, and may be held by re-election for a further period of three or six months, but no longer. No salary attached to the posts, but residence, board, and washing will be provided. Applications and testimonials to Howard J. Collins, House Governor.

Joint Counties Lunatic Asylum, Carmarthen. Medical Superintendent. Salary £500 per annum, with unfurnished house, garden produce, fire, light, and washing. Applications and testimonials to be forwarded to W. Morgan Griffiths, Solicitor, Carmarthen, by March 24th.

Taunton and Somerset Hospital. Assistant House-Surgeon. Appointment for six months. No salary, but board, washing, and lodging in the institution. Applications and testimonials to the House Surgeon by March 24th.

#### Non-Resident Medical Officers.

County of Northumberland. Medical Officer of Health. Salary £500 per annum, with travelling expenses. Appointment for three years. Applications and testimonials, endorsed "Medical Officer," to C. D. Forster, Clerk to the Council, by March 24th.

Hospital for Sick Children, Great Ormond Street, Bloomsbury, W.C. House Surgeon to Out-patients. Appointment for six months, but the holder will be eligible for a second term of office. Salary 25 guineas. Applications and testimonials to Adrian Hope, Secretary, by March 20th.

Infirmary for Consumption and Diseases of the Chest and Throat, 26, Margaret Street, Cavendish Square, W. Honorary Visiting Physician; must reside within one mile of the Institution. Particulars of qualifications to be obtained at the Infirmary.

Owen College, Manchester. Professor of Zoology. Applications to the Council of the College, under cover to the Registrar, by April 3rd.

Royal College of Physicians. Milroy Lecturer. Applications to Edward Liveing, M.D., Registrar, by April 9th.

West End Hospital for Nervous Diseases, &c., 73, Welbeck Street, W. Anæsthetist. Appointment for twelve months. Candidate eligible for re-election. Applications to H. Ansell, Secretary.

### Books Received.

David Stott.

"Character and Fortune Revealed: An A B C Guide to Palmistry." By Paul Bello.

Longmans, Green, and Co

"Dr. Chesterfield's Letters to his Son on Medicine as a Career." By Sir William B. Dalby. Price 1s.

"A Gauntlet, being the Norwegian Drama 'En Hanske." By Björnstjerne Björnson, translated into English by Osman Edwards.

W. B. Saunders, Philadelphia.

"A Text-Book of the Theory and Practice of Medicine, by American Teachers." Edited by William Pepper, M.D., LL.D. Vol. II.

V. Kirchbaum, St. Petersburg.

"Bulletin Russe de Statistique Financière, et de Législation." 1ère année.

A. and C. Black.

"Alladdin in London." By Fergus Hume.

"The Story of a Struggle." By Elizabeth Gilkison.

G. Warne and Co.

"In Love with the Czarina." By S. Jokai.

Hirschfeld Brothers.

"Food and Drink Rationally Discussed." By Thomas Dutton, M.D., Univ. Durh.

Henry Kimpton.

"A Short Guide to the Examination of Lying-in Women." By Professor Credé and Professor Leopold. Translated by William H. Wilson, M.A., M.D. (Oxon).

Scientific Press.

"Burdett's Hospital and Charities Annual, 1894." Edited by Henry C. Burdett.

Iliffe and Son.

"The Cyclist Year Book, 1891." Edited by Henry Sturmey.

**Periodicals and Pamphlets.**—Cassel's Family Magazine; the Cornhill Magazine; the Humanitarian; the Westminster Review; the Ludgate Monthly; Essays on State Medicine (1)—Compulsory Vaccination; Leeds Hospital Magazine; Children's League of Pity Paper; Toilers of the Deep; In His Name; Reich's Medicinal Anzeiger.

